# Multi-Locus Phylogeny and Taxonomy of the Fungal Complex Associated With Rusty Root Rot of *Panax ginseng* in China

**DOI:** 10.3389/fmicb.2020.618942

**Published:** 2020-12-16

**Authors:** Yi Ming Guan, Ying Ying Ma, Qiao Jin, Qiu Xia Wang, Ning Liu, Yong Ping Fu, Ya Yu Zhang, Yu Li

**Affiliations:** ^1^Engineering Research Center, Chinese Ministry of Education for Edible and Medicinal Fungi, Jilin Agricultural University, Changchun, China; ^2^Institute of Special Wild Economic Animal and Plant Science, Chinese Academy of Agricultural Sciences, Changchun, China

**Keywords:** *Dactylonectria*, *Ilyonectria*, *Ilyonectria*-like, *Neonectria*, *Panax ginseng*, *Rhexocercosporidium*, rusty root rot, *Thelonectria*

## Abstract

Panax *ginseng* rusty root rot caused by the *Ilyonectria* species complex is a devastating disease, and it is one of the main factors contributing to the difficulty in continual cropping. Rusty root rot occurs in all ginseng fields, but little is known about the taxonomy of the fungal pathogen complex, especially *Ilyonectria* and *Ilyonectria*-like species. Rusty root rot samples were collected from commercial ginseng cultivation areas of China, and the pathogens were isolated and purified as single spores. Based on the combination analysis of multiple loci (rDNA-ITS, *TUB*, *HIS3*, *TEF*, *ACT*, LSU, *RPB1*, *RPB2*, and SSU) and morphological characteristics, the pathogens causing ginseng rusty root rot were determined. Fungal isolates were obtained from infected roots in 56 locations within main cultivation areas in China. A total of 766 strains were identified as *Ilyonectria*, *Ilyonectria*-like and *Rhexocercosporidium* species, including *I. robusta* (55.0%), *I. communis* (21.7%), *I. mors-panacis* (10.9%), *I. pseudodestructans* (2.0%), *I. changbaiensis* (1.3%), *I. qitaiheensis* (1.3%), *Neonectria obtusispora* (2.0%), *Dactylonectria torresensis* (0.5%), *D.* sp. (0.5%), and *R. panacis* (1.5%), and four novel species, *Thelonectria ginsengicola* (1.0%), *T. jixiensis* (1.0%), *T. mulanensis* (0.8%) and *T. fusongensis* (0.5%), with a total of 14 species. As the pathogen present in the highest proportion, *I. robusta* was the most prevalent and damaging species, unlike the pathogens reported previously. All of the examined strains were proven to cause ginseng rusty root rot. Our results indicate that the taxonomy of the fungal complex associated with ginseng rusty root rot includes *Ilyonectria*, *Ilyonectria*-like genera (*Dactylonectria*, *Neonectria*, and *Thelonectria*) and *Rhexocercosporidium*.

## Introduction

*Panax ginseng* is one of the most cultivated medicinal plants in China, and the quality of ginseng greatly influences the quality of cosmetics, health care products and medicines that use ginseng as a raw material (Baranov, 1966; Hu, 1977). Ginseng rusty root rot is caused by *Ilyonectria*/*Cylindrocarpon* or *Ilyonectria*/*Cylindrocarpon*-like species, and it is the most devastating chronic disease and the greatest threat to ginseng (*Panax ginseng*) cultivation (Guan et al., 2020). Rusty root rot may cause 30% loss of ginseng, and it can be found in all ginseng planting areas. Rusty root rot has always been one of the important factors that interferes with the maintenance of continuous ginseng cropping. The incidence of rusty root rot in 2-year-old ginseng continuous cropping is 95.8% (Cho et al., 1995).

All parts of the ginseng root may be infected, and the infected root exhibits reddish brown dry rot with a gully appearing on most of the root. The infection stops with increased temperature, and some parts of the ginseng root may undergo self-healing, but the gully or dry rot scars remain. The appearance of ginseng root rot and the degree of damage greatly influence the value of ginseng (Zhou et al., 2017). *Ilyonectria* and *Ilyonectria*-like species are common soil fungi, opportunistic plant root pathogens, or asymptomatic root endophytes (Seifert et al., 2003). Ginseng seeds also carry *Ilyonectria* pathogens that may pose a threat to ginseng cultivation (Guan et al., 2019). *Ilyonectria* fungi play an important role in black foot rot in grapevines (Halleen et al., 2004; Halleen et al., 2006a,b), apple replant disease (Tewoldemedhin et al., 2011), and beech cankers (Castlebury et al., 2006); the affected plants are representative hosts of economic importance, especially the lignified roots of perennials. Thus, *Ilyonectria* and *Ilyonectria*-like species are commonly associated with rot and decay of woody and herbaceous plants (Domsch et al., 2007).

The pathogen associated with root rot disease of *Panax quinguefaliam* was first extracted by Zinnsmeister (1918), and it was designated *Ramularia destructans*. Similar diseases were found by Chou and Chung on ginseng in China and Korea (Chung, 1979; Wang, 2001). Scholten established the new species *C. destructans*, and *R. destructans* was treated as a synonym (Scholten, 1964).

*Neonectria*/*Cylindrocarpon* is a paraphyletic based on phylogenetic analysis (Mantiri et al., 2001; Halleen et al., 2004; Hirooka et al., 2005; Castlebury et al., 2006). The *Neonectria* complex was divided into four genera (*Ilyonectria*, *Neonectria*/*Cylindrocarpon*, *Rugonectria* and *Thelonectria*) based on a combination of morphological characteristics. *Ilyonectria* replaced the *N. radicicola* group based on the description by Booth (1966); Seifert et al. (2003), and the corresponding *Cylindrocarpon* also became *Ilyonectria* (Mantiri et al., 2001).

*I. destructans* was linked to the teleomorph *I. radicicola* (Booth, 1966; Samuels and Brayford, 1990; Chaverri et al., 2011). Based on a phylogenetic analysis of nuclear ribosomal internal transcribed spacer (rDNA-ITS) gene sequences, Schroers et al. concluded that the *I. radicicola* complex included *C. destructans*, *C. destructans var. crassum, I. coprosmae, I. liriodendri, N. austroradicicola* and *N. macroconidialis* (Schroers et al., 2007). The rDNA-ITS, β-tubulin (*TUB*), histone H3 (*HIS3*) and translation elongation factor 1-α (*TEF*) genes were used to support the *I. radicicola* species complex, and *HIS3* was the most contributing gene (Cabral et al., 2012b). Sixty-eight strains of pathogenic fungi from ginseng and other hosts were once considered *C. destructans* within four groups: *I. mors-panacis*, *I. robusta*, *I. panacis*, and *I. crassa* (Cabral et al., 2012a). Lu et al. (2020) obtained 230 isolates and believed that ginseng red-sin root was caused by 12 species, including *Fusarium*, *Dactylonectria*, *Ilyonectria* and others.

*Thelonectria* was first classified in *Cylindrocarpon* and first described in 2011 to accommodate grouping in the *Cylindrocarpon* classification (Chaverri et al., 2011). However, the forms isolated from soil were the asexual states of *Thelonectria*. *Thelonectria* and related species with *Cylindrocarpon*-like morphology were redefined using molecular phylogenetic techniques (Chaverri et al., 2011; Salgado-Salazar et al., 2012, 2013, 2015). The observation that *R. panacis* causes ginseng rusted root rot was reported for the first time in 2006, after more than 70 years of the disease (Reeleder et al., 2006; Reeleder, 2007).

In this work, we report the population structure of pathogens associated with ginseng rusty root rot. In the commercial ginseng cultivation areas in China, 90% of rusty root rot is thought to be caused by *I. destructans* (Wang, 2001), but the experimental data support different conclusions. The present study determined that the pathogens that cause rusty root rot disease of ginseng are consistently associated with a fungal complex species, and management strategies must be developed.

## Materials and Methods

### Sample Collection, Fungal Isolation, and Morphological Observation

**FIGURE 1:**
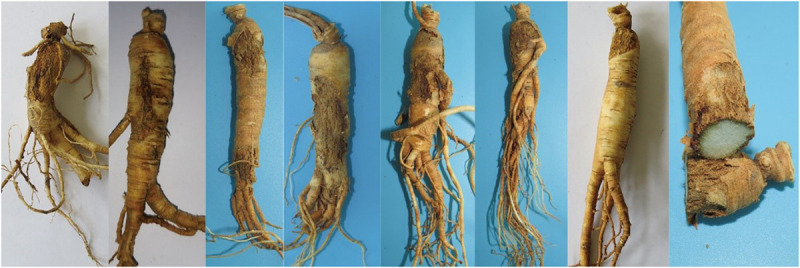
Symptoms of *Panax ginseng* rusty root rot disease.

**FIGURE 2:**
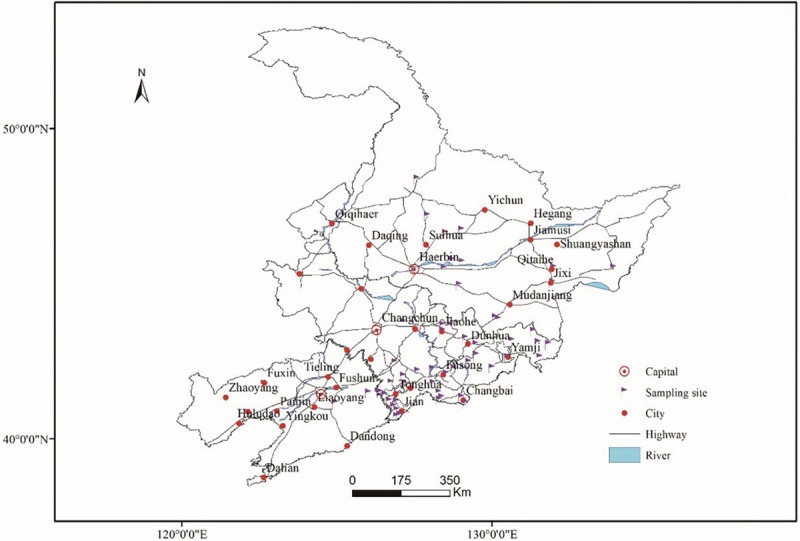
Sampling locations in the main ginseng cultivation areas of China.

Fresh ginseng roots with rusty root rot symptoms (Figure 1) were collected between 2017 and 2019 from 56 sampling locations in northeast China that corresponded to the commercial ginseng-producing areas of China (Figure 2). The junction of the healthy and diseased parts was cut into 25-mm^3^ pieces, and the surfaces of the pieces were disinfected by immersion in 1% NaOCl for 2 min and washing three times with sterile water. The pieces were evenly placed on water agar (WA) medium and cultured in the dark at 22°C. Colonies were collected for 15-21 days, purified and cultured from single spores (Punja, 1997). The cultures were transferred to potato dextrose agar (PDA) medium and synthetic nutrient-poor agar (SNA) and cultured in the dark for 20-60 days, and the characteristics of the resulting cultures were recorded. The isolates were cultivated on PDA supplemented with ginseng roots under continuous near-ultraviolet light (n-UV, 315-400 nm), and the characteristics of conidiophore cells and the shape and size of chlamydospores and conidia were observed. Images and measurements were acquired using a KEYENCE VXH-5000 digital microscope system (Osaka, Japan).

### DNA Extraction and PCR Amplification

Colonies grown for 15 days were collected for DNA extraction. DNA was obtained by following the procedures described in the Qiagen DNeasy Plant Mini Kit (Qiagen 69104, Qiagen, Hilden, Germany). Each 50-μl PCR aliquot contained 25 ml of premix (Takara R045, Kusatsu, Japan; the error rate of the DNA polymerase is 0.02%), 2 μl of each primer (10 μM), 1 μl of DNA (500 ng/μl), and 20 μl of double-distilled H_2_O. The following PCR program was used to obtain the sequences of the actin gene (*ACT*), rDNA-ITS, the 28S ribosomal RNA gene (LSU), the gene encoding RNA polymerase II largest subunit (*RPB1*), the gene encoding RNA polymerase II second-largest subunit (*RPB2*), 18S ribosomal RNA (SSU), *TEF*, *TUB* and *HIS3*: 95°C for 3 min; 30 cycles of 95°C for 15 s, annealing temperature for 15 s, 72°C for 60 s, and 72°C for 10 min; the sample was then held at 4°C. The amplicons were analyzed using 0.8% agarose gel electrophoresis in 0.5 × TAE buffer (150 V for 25 min) and purified using the MinElute PCR Purification Kit (Qiagen 28004, Qiagen, Hilden, Germany). Details regarding the PCR primers and annealing temperatures are listed in Table 1.

**TABLE 1:** Details regarding the PCR primers used in species identification and gene sequencing.

**Gene region**	**Primer**	**Direction**	**Annealing temperature (°C)**	**References**
Nuclear ribosomal internal transcribed spacer	V9G	sense	51	de Hoog and van den Ende, 1998
	ITS4	antisense		
Actin	ACT512F	sense	51	Carbone and Kohn, 1999; Groenewald et al., 2013
	ACT1RD	antisense		
28S ribosomal RNA gene	LROR	sense	48	Vilgalys and Hester, 1990; Moncalvo et al., 2000
	LR5	antisense		
RNA polymerase II largest subunit	RPB1a	sense	55	Castlebury et al., 2004
	RPB1c	antisense		
Histone H3 gene	CYLH3F	sense	50	Crous et al., 2004
	CYLH3R	antisense		
RNA polymerase II second-largest subunit	RPB2-7cf	sense	54	Liu et al., 1999
	RPB2-11aR	antisense		
18S ribosomal RNA gene	NS1	sense	55	Gargas and Taylor, 1992
	SR7	antisense		
β-tubulin	BT3	sense	52	Lu et al., 2020; O’Donnell and Cigelnik, 1997
	BT4	antisense		
	T1	sense		
	T2	antisense		
Translation elongation factor 1-alpha	CYLEF-1	sense	55	Carbone and Kohn, 1999; Crous et al., 2004
	CYLEF-R2	antisense		
	EF728	sense		
	EF1567	antisense		

### DNA Sequencing and Phylogenetic Analysis

The purified amplicons were sequenced in both directions by Sangon Biotech (Shanghai, China), and the sequences were assembled using Seqman (v8.1). Phylogenetic trees were constructed using the combined rDNA-ITS, *TUB*, *HIS3* and *TEF* genes for analyses of *Dactylonectria*, *Ilyonectria* and *Neonectria*, and the analysis of *Thelonectria* was based on a combination of the *ACT*, rDNA-ITS, *LSU*, *RPB1*, *RPB2*, SSU, *TEF* and *TUB* genes. All sequences were homologously aligned by mega 7.06. A maximum parsimony (MP) method was used to construct a phylogenetic tree in the PAUP 4.0b program. The MP analyses were performed with the heuristic search option, and 100 random sequence additions were used to find the global optimum tree. The gaps were treated as missing data, and the strength of the internal branches of the resulting trees was tested with bootstrap analysis using 1000 replications. The consistency index (CI), retention index (RI), and rescaled consistency index (RC) of the tree were also calculated. Detailed information on the sequences of the genes whose accession numbers were applied for in the NCBI database, including all strains used for phylogeny, is shown in Tables 2, 3.

**TABLE 2:** Strains analyzed in this study.

**Species**	**Strain^*a*^**	**Origin**	**GenBank accession number^*b*^**								
			**rDNA-ITS**	***TUB***	***HIS3***	***TEF1-α***	***ACT***	**LSU**	***RPB1***	***RPB2***	**SSU**
*D. alcacerensis*	IAFM Cy20-1	Spain	JF735332	AM419104	JF735629	JF735818					
*D. alcacerensis*	129087	Portugal	JF735333	AM419111	JF735630	JF735819					
*D. anthruriicola*	CBS 564.95	Netherlands	JF735302	JF735430	JF735579	JF735768					
*D. estremocensis*	Cy135	Portugal	AM419069	AM419105	JF735615	JF735804					
*D. estremocensis*	CBS 129085	Portugal	JF735320	JF735448	JF735617	JF735806					
*D. hordeicola*	CBS 162.89	Netherlands	AM419060	AM419084	JF735610	JF735799					
*D. hordeicola*	3S07	China	MF350482	MF350428	MF350455	MF350509					
*D. macrodidyma*	CBS 112615	South Africa	AY677290	AY677233	JF735647	JF735836					
*D. macrodidyma*	CBS 112601	South Africa	AY677284	AY677229	JF735644	JF735833					
*D. novozelandica*	CBS 112608	South Africa	AY677288	AY677235	JF735632	JF735821					
*D. novozelandica*	CBS 113552	New Zealand	JF735334	AY677237	JF735633	JF735822					
*D. pinicola*	CBS 159.34	UK: England	JF735318	JF735446	JF735613	JF735802					
*D. pinicola*	CBS 173.37	Germany	JF735319	JF735447	JF735614	JF735803					
*D.* sp.	Q18-22	China	MT678572*	MT810745*	MT800956*	MT800973*					
*D.* sp.	Q18-23	China	MT678573*	MT810746*	MT800957*	MT800974*					
*D. torresensis*	CBS 129086	Portugal	JF735362	JF735492	JF735681	JF735870					
*D. torresensis*	CBS 113555	New Zealand	JF735350	AY677234	JF735661	JF735850					
*D. torresensis*	DT2	China	MT678571*	MT810744*	MT800955*	MT800972*					
*D. vitis*	CBS 129082	Portugal	JF735303	JF735431	JF735580	JF735769					
*I. changbaiensis*	CGMCC 3.18789	China	MF350464	MF350410	MF350437	MF350491					
*I. changbaiensis*	72R2	China	MF350465	MF350411	MF350438	MF350492					
*I. changbaiensis*	CB4-7	China	MT678567*	MT810740*	MT800951*	MT800968*					
*I. changbaiensis*	Q24-5	China	MT678568*	MT810741*	MT800952*	MT800969*					
*I. communis*	CGMCC 3.18788	China	MF350402	MF350402	MF350429	MF350483					
*I. communis*	J410	China	MF350457	MF350403	MF350430	MF350484					
*I. communis*	CB4-2	China	MT678565*	MT810738*	MT800949*	MT800966*					
*I. communis*	H1-9	China	MT678566*	MT810739*	MT800950*	MT800967*					
*I. coprosmae*	CBS 119606	Canada	JF735260	JF735373	JF735505	JF735694					
*I. crassa*	CBS 129083	Canada	AY295311	JF735395	JF735536	JF735725					
*I. crassa*	CBS 158.31	JF735694	JF735276	JF735394	JF735535	JF735724					
*I. cyclaminicola*	CBS 302.93	Netherlands	JF735304	JF735432	JF735581	JF735770					
*I. cyclaminicola*	EFA-444	Spain	MF440369	MF797792	MF471472	MH070096					
*I. destructans*	CBS 264.65	Sweden	AY677273	AY677256	JF735506	JF735695					
*I. europaea*	CBS 102892	Germany	JF735295	JF735422	JF735569	JF735758					
*I. europaea*	CBS 129078	Portugal	JF735294	JF735421	JF735567	JF735756					
*I. gamsii*	CBS 940.97	Netherlands	AM419065	AM419089	JF735577	JF735766					
*I. leucospermi*	CBS 13289	South Africa	JX231161	JX231113	JX231145	JX231129					
*I. leucospermi*	CBS 132810	South Africa	JX231162	JX231114	JX231146	JX231130					
*I. liliigena*	CBS 732.74	Netherlands	JF735298	JF735426	JF735574	JF735763					
*I. liliigena*	CBS 189.49	Netherlands	JF735297	JF735425	JF735573	JF735762					
*I. liriodendri*	CBS 117526	Portugal	DQ178164	DQ178171	JF735508	JF735697					
*I. liriodendri*	CBS 110.81	United States	DQ178163	DQ178170	JF735507	JF735696					
*I. lusitanica*	CBS 129080	Portugal	JF735296	JF735423	JF735570	JF735759					
*I. mors-panasis*	CBS 306.35	Canada	JF735288	JF735414	JF735557	JF735746					
*I. mors-panasis*	H6-1	China	MT678563*	MT810736*	MT800947*	MT800964*					
*I. mors-panasis*	XFC1	China	MT678564*	MT810737*	MT800948*	MT800965*					
*I. panasis*	CBS 129079	Canada	AY295316	JF735424	JF735572	JF735761					
*I. palmarum*	CBS 135753	Italy	HF937432	HF922609	HF922621	HF922615					
*I. palmarum*	CBS 135754	Italy	HF937431	HF922608	HF922620	HF922614					
*I. protearum*	CBS 132812	South Africa	JX231165	JX231117	JX231149	JX231133					
*I. protearum*	CBS 132811	South Africa	JX231157	JX231109	JX231141	JX231125					
*I. pseudodestructans*	CBS 129081	Portugal	AJ875330	AM419091	JF735563	JF735752					
*I. pseudodestructans*	CBS 117824	Austria	JF735292	JF735419	JF735562	JF735751					
*I. pseudodestructans*	ZP2	China	MT678561*	MT810734*	MT800945*	MT800962*					
*I. pseudodestructans*	PR20-11	China	MT678562*	MT810735*	MT800946*	MT800963*					
*I. qitaiheensis*	CGMCC 3.18787	China	MF350472	MF350418	MF350445	MF350499					
*I. qitaiheensis*	J919	China	MF350473	MF350419	MF350446	MF350500					
*I. qitaiheensis*	R3-2	China	MT678569*	MT810742*	MT800953*	MT800970*					
*I. qitaiheensis*	PR14 25	China	MT678570*	MT810743*	MT800954*	MT800971*					
*I. robusta*	CBS 117818	Austria	JF735267	JF735382	JF735523	JF735712					
*I. robusta*	CBS 129084	Portugal	JF735273	JF735391	JF735532	JF735721					
*I. robusta*	H8-5	China	MT678559*	MT810732*	MT800943*	MT800960*					
*I. robusta*	CB13-12	China	MT678560*	MT810733*	MT800944*	MT800961*					
*I. rufa*	CBS 153.37	France	AY677271	AY677251	JF735540	JF735729					
*I. rufa*	CBS 640.77	France	JF735277	JF735399	JF735542	JF735731					
*I. g*	CBS 142253	Italy	KY304649	KY304755	KY304621	KY304727					
*I. strelitziae*	CBS 142254	Italy	KY304651	KY304757	KY304623	KY304729					
*I. vredehoekensis*	CBS 132807	South Africa	JX231155	JX231107	JX231139	JX231123					
*I. vredehoekensis*	CBS 132808	South Africa	JX231159	JX231111	JX231143	JX231127					
*N. coccinea*	CBS 119158	Germany	JF268759	KC660727		JF268734					
*N. faginata*	CBS 217.67	Canada	HQ840385	JF268730		JF268746					
*N. faginata*	CBS 119160	United States	HQ840384	DQ789883		DQ789740					
*N. lugdunenis*	CBS 1254585	China	KM231762	KM232019	KM231482	KM231187					
*N. obtusispora*	CBS 183.36	Germany	AM419061	AM419085	JF735607	JF735796					
*N. obtusispora*	CPC 13544	Canada	AY295306	JF735443	JF735608	JF735797					
*N. obtusispora*	H7-6	China	MT678574V	MT810747*	MT800958*	MT800975*					
*N. obtusispora*	Q12-4	China	MT678575*	MT810748*	MT800959V	MT800976*					
*N. punicea*	CBS 242.29	Germany	KC660522	DQ789873		DQ789730					
*N. punicea*	CBS 119724	Austria	KC660469	DQ789824		KC660431					
*N. ramulariae*	CBS 151.29	England	AY677291	JF735438	JF735602	JF735791					
*N. ramulariae*	CBS 182.36	Unknown	HM054157	JF735439	JF735603	JF735792					
*N. shennongjiana*	CBS 127475	China	MH864598	KJ022346		KJ022406					
*N. shennongjiana*	HMAS 183185	China	FJ560440	FJ860057							
*Rhexocercosporidium panacis*	RP17	China	MT814852*	MT822282*	MT822281*						
*T. acrotyla*	IMI 345086	Venezuela	KJ021971	KJ022293		KJ022347	KJ022238	KJ022026	KJ022407	KJ022590	KJ022212
*T. acrotyla*	CBS 123766	Venezuela	JQ403329	JQ394720		JQ394751	JQ365047	JQ403368	JQ403407		
*T. amamiensis*	MAFF 239820	Japan	JQ403338	JQ394728		KJ022349	JQ365055	JQ403376	JQ403413	KJ022595	KJ022216
*T. amamiensis*	MAFF 239819	Japan	JQ403337	JQ394727		KJ022348	JQ365054	JQ403375	KJ022408	KJ022594	KJ022215
*T. blackeriella*	CBS 142200	Italy	KX778711	KX778702			KX778687	KX778690	KX778693		
*T. cidaria*	CBS 132324	Costa Rica	KJ021972	JQ394715		KJ022351	JQ365043	JQ403324	JQ403402	KJ022508	KJ022153
*T. cidaria*	IMI 325844	Jamaica	KJ021973	JQ394707		JQ394741	KJ022239	KJ022027	JQ403392	KJ022508	KJ022130
*T. coronalis*	CBS 132337	China	JQ403343	JQ394732		JQ394761	KJ022240	MH877458	JQ403418	KJ022459	KJ022080
*T. coronalis*	CBS 132338	China	JQ403344	JQ394733		KJ022352	KJ022241	JQ403381	JQ403419	KJ022462	KJ022083
*T. coronata*	IMI 325241	Indonesia	JQ403326	JQ394717		KJ394749	JQ365044	JQ403365	JQ403404	KJ022164	KJ022542
*T. coronata*	CBS 132322	Costa Rica	JQ403320	JQ394711		JQ294736	JQ365040	JQ403360	JQ403397	KJ022521	KJ022523
*T. diademata*	CBS 132331	Argentina	JQ403308	JQ394700		JQ394736	JQ365029	JQ403308	JQ403384	KJ022099	KJ022474
*T. diademata*	CBS 132332	Argentina	JQ403351	KJ022321		KJ022383	JQ365032	JQ403311	KJ403387	KJ022099	KJ022478
*T. gongylodes*	CBS 124611	United States	JQ403318	JQ394710		JQ394744	JQ365038	HQ403358	JQ403395	KJ022514	KJ022136
*T. gongylodes*	IMI 343571	United States	JQ403331	JQ394721		JQ394752	JQ365048	JQ403370	JQ403408	KJ022564	KJ022186
*T. nodosa*	CBS 124742	United States	JQ403306	JQ394699		JQ394735	JQ365028	JQ403346	JQ403383	KJ022469	KJ022090
*T. nodosa*	CBS 132327	United States	JQ403317	JQ394709		JQ394743	JQ365037	JQ403357	JQ403394	KJ022513	KJ022135
*T. olida*	CBS 215. 67	Germany	KJ021982	KM232024			HM352884	KJ022058	HM364334	KM232342	
*T. stemmata*	CBS 112468	Jamaica	JQ403312	JQ394704		JQ394739	JQ365033	JQ403352	JQ403388	KJ022502	KJ022124
*T. stemmata*	CBS 132336	Jamaica	JQ403313	JQ394705		KJ022384	JQ365034	JQ403353	JQ403389	KJ022503	KJ022125
*T. torulosa*	CBS 136782	Cameroon	KJ022007			KJ022390	KJ022274	KJ022038	KJ022438	KJ022519	KJ022141
*T. torulosa*	CBS 132339	Argentina	JQ403309	JQ394701		KJ022389	JQ365030	JQ403349	JQ403385	KJ022473	KJ022094
*T. truncata*	MAFF 241521	Japan	JQ403339	KJ022325		JQ394757	JQ365056	JQ403377	JQ403414	KJ022601	KJ022222
*T. veuillotiana*	CBS 132341	Azores Island	JQ403305	JQ394698		JQ394734	KJ022273	JQ403345	JQ403382	KJ022465	KJ022086
*T. veuillotiana*	CBS 124114	France	JQ403335	JQ394725		JQ394755	GQ505980	GQ506005	GQ506034	KJ022568	KJ022190
*T. westlandica*	ICMP 10387	New Zealand	KF569844	KJ569871		KF569861	KF569834	KF569852	KF569880	KJ022577	KJ022199
*T. westlandica*	IMI 255610	New Zealand	KF569843	KF569870		KF569862	KF569833	KF569853	KF569881	KJ022581	KJ0222033
*T. ginsengcola*	CGMCC 3.20154; R9	China	MT742968*	MT792252*		MT792268*	MT792260*	MT742978*	MT792236*	MT792244*	MT742994*
*T. ginsengcola*	R4	China	MT742969*	MT792253*		MT792269*	MT792261*	MT742979*	MT792237*	MT792245*	MT742995*
*T. mulanensis*	CGMCC 3.20155; Q20-8	China	MT742970*	MT792254*		MT792270*	MT792262*	MT742980*	MT792238*	MT792246*	MT742996*
*T. mulanensis*	Q20-5	China	MT742971*	MT792255*		MT792271*	MT792263*	MT742981*	MT792239*	MT792247*	MT742997*
*T. fusongensis*	CGMCC 3.20153; R1-8	China	MT742972*	MT792256*		MT792272*	MT792264*	MT742982*	MT792240*	MT792248*	MT742998*
*T. fusongensis*	P4	China	MT742973*	MT792257*		MT792273*	MT792265*	MT742983*	MT792241*	MT792249*	MT742999*
*T. jixiensis*	CGMCC 3.20156; Q21-5	China	MT742974*	MT792258*		MT792274*	MT792266*	MT742984*	MT792242*	MT792250*	MT743000*
*T. jixiensis*	Q21-1	China	MT742975*	MT792259*		MT792275*	MT792267*	MT742985*	MT792243*	MT792251*	MT743001*
*Cinnmomeonectria cinammomea*	CBS 133756	French Guiana	KJ021979	KJ022341		KJ022393	KJ0221286	KJ022072	KJ022451	KJ022584	KJ022206

^*a*^IAFM, Instituto Agroforestal Mediterráneo, Universidad Politécnica de Valencia, Spain; CBS, culture collection of the Centraalbureau voor Schimmelcultures, Fungal Biodiversity Centre, Utrecht, Netherlands; CPC, Culture collection of Pedro Crous, housed at CBS; ICMP, International Collection of Microorganisms from Plants, Auckland, New Zealand; IMI, International Mycological Institute, CABI-Bioscience, Egham, Bakeham Lane, United Kingdom; CGMCC, China General Microbiological Culture Collection Center, Beijing, China. ^*b*^rDNA-ITS, Nuclear ribosomal internal transcribed spacer; *HIS3*, histone H3 gene; *TEF-1a*, translation elongation factor 1-alpha gene; *TUB*, β-tubulin gene; *ACT*, actin gene; LSU, 28S ribosomal RNA gene; *RPB1*, RNA polymerase II largest subunit gene; *RPB2*, RNA polymerase II second-largest subunit gene; SSU, 18S ribosomal RNA gene. *Sequences newly generated in this study.

**TABLE 3:** Detailed information on the collection of isolates used in sequencing and pathogenicity testing.

**Species**	**Strain**	**Origin**	**Collection date**	**Host**	**Years**
*D.* sp.	Q18-22	Yongqing Town, Antu City, Jilin Province, China	08, 2019	*P. ginseng*	3
*D.* sp.	Q18-23	Xinhe Town, Antu City, Jilin Province, China	09, 2018	*P. ginseng*	4
*D. torresensis*	DT2	Duling Town, Tonghua City, Jilin Proinvce, China	06, 2017	*P. ginseng*	4
*I. changbaiensis*	CB4-7	Beigang Town, Baishan City, Jilin Province, China	07, 2019	*P. ginseng*	4
*I. changbaiensis*	Q24-5	Datougou Town, Wangqing City, Jilin Province, China	08, 2018	*P. ginseng*	3
*I. communis*	CB4-2	Dongbeicha Town, Baishan City, Jilin Province, China	08, 2018	*P. ginseng*	3
*I. communis*	H1-9	Hulin Town, Jixi City, Heilongjiang Province, China	09, 2017	*P. ginseng*	4
*I. mors-panasis*	H6-1	Hailun Town, Hailun City, Heilongjiang Province, China	09, 2019	*P. ginseng*	5
*I. mors-panasis*	XFC1	Xigang Town, Baishan City, Jilin Province, China	06, 2018	*P. ginseng*	3
*I. pseudodestructans*	ZP2	Zuojia Town, Jili City, Jilin Province, China	06.2018	*P. ginseng*	4
*I. pseudodestructans*	PR20-11	Datougou Town, Wangqing City, Jilin Province, China	06, 2018	*P. ginseng*	5
*I. qitaiheensis*	R3-2	Longquan Town, Baishan City, Jilin Province, China	06, 2018	*P. ginseng*	3
*I. qitaiheensis*	PR14 25	Fusong County, Baishan City, Jilin Province, China	06, 2018	*P. ginseng*	3
*I. robusta*	H8-5	Hailin Town, Mudanjiang City, Heilongjiang Province, China	08, 2019	*P. ginseng*	4
*I. robusta*	CB13-12	Wanliang Town, Fusong County, Baishan City, Jilin Province, China	08, 2019	*P. ginseng*	2
*N. obtusispora*	H7-6	Zhanhe Town, Heihe City, Heilongjiang	08, 2019	*P. ginseng*	3
*N. obtusispora*	Q12-4	Longquan Town, Baishan City, Jilin Province, China	08, 2019	*P. ginseng*	4
*Rhexocercosporidium panacis*	RP17	Yulin Town, Jian City, Jilin Province, China	08, 2019	*P. ginseng*	3
*T. ginsengcola*	CGMCC 3.20154; R9	Dongbeicha Town, Baishan City, Jilin Province, China	07, 2018	*P. ginseng*	4
*T. ginsengcola*	R4	Quanyang Town, Baishan City, Jilin Province, China	07, 2018	*P. ginseng*	3
*T. mulanensis*	CGMCC 3.20155; Q20-8	Bayan Town, Mulan County, Heilongjiang, China	06, 2017	*P. ginseng*	4
*T. mulanensis*	Q20-5	Mulan Town, Mulan County, Heilongjiang, China	06, 2017	*P. ginseng*	4
*T. fusongensis*	CGMCC 3.20153; R1-8	Fusong Town, Fusong County, Baishan City, Jilin Province, China	06, 2017	*P. ginseng*	3
*T. fusongensis*	P4	Wanliang Town, Fusong County, Baishan City, Jilin Province, China	06, 2017	*P. ginseng*	3
*T. jixiensis*	CGMCC 3.20156; Q21-5	Hulin Town, Jixi City, Heilongjiang Province, China	08, 2018	*P. ginseng*	4
*T. jixiensis*	Q21-1	Hulin Town, Jixi City, Heilongjiang Province, China	07, 2018	*P. ginseng*	3

### Pathogenicity Tests

The 26 newly sequenced strains were also subjected to pathogenicity testing (Table 3). The pathogenicity of the isolates was evaluated on 3-year-old detached ginseng roots (cultivar: Damaya) using an improved published method *in vitro* (Lu et al., 2020). The fresh ginseng roots were washed, wiped with 75% alcohol, and rinsed with sterile water. Healthy ginseng roots were inoculated by placing 50 μl of 1 × 10^5^/ml spore suspension taken from the edges of actively growing colonies on PDA plates into premade holes 4 mm in diameter and 1 mm deep. Two to three holes per root and four replicated roots were inoculated for each isolate, and sterile water was used as the control. The ginseng roots were cultured in a fresh-keeping box at 25°C in the dark and evaluated after 7 days. The same strain and disinfection method were used to inoculate whole plants in the greenhouse (25°C, 78% humidity). After disinfection, 3-year-old ginseng seedlings were dipped into a 1 × 10^5^/ml spore suspension for 20 min and then transferred to pots containing sterile sand, with 6 plants per pot. Each group was grown in triplicate. The inoculum was treated with sterile water as a control. After 2 months, plant infection was evaluated.

## Results

### Population Structure of Fungal Complex Associated With Ginseng Rusty Root Rot

**FIGURE 3:**
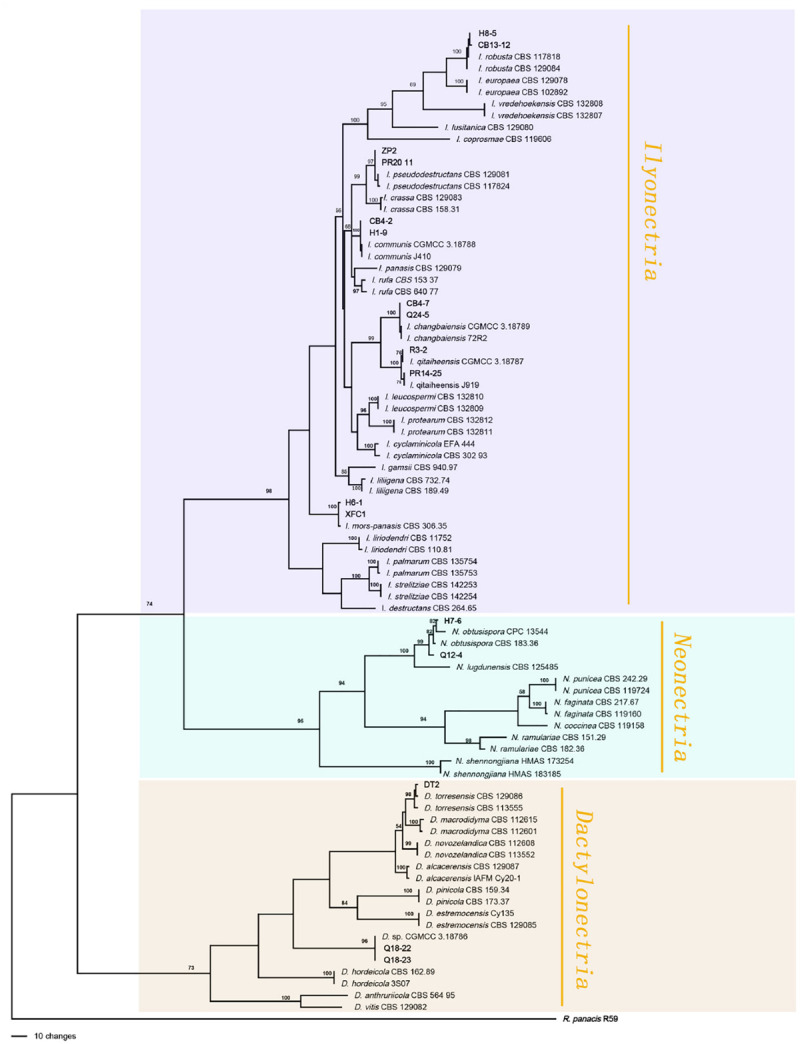
Phylogenetic tree based on the combined rDNA-ITS, *TUB*, *HIS3* and *TEF* gene sequences constructed using the maximum parsimony method of the PAUP 4.0b program. *Rhexocercosporidium panacis* R59 was used as an outgroup. Bold font indicates the strains isolated in this study.

**FIGURE 4:**
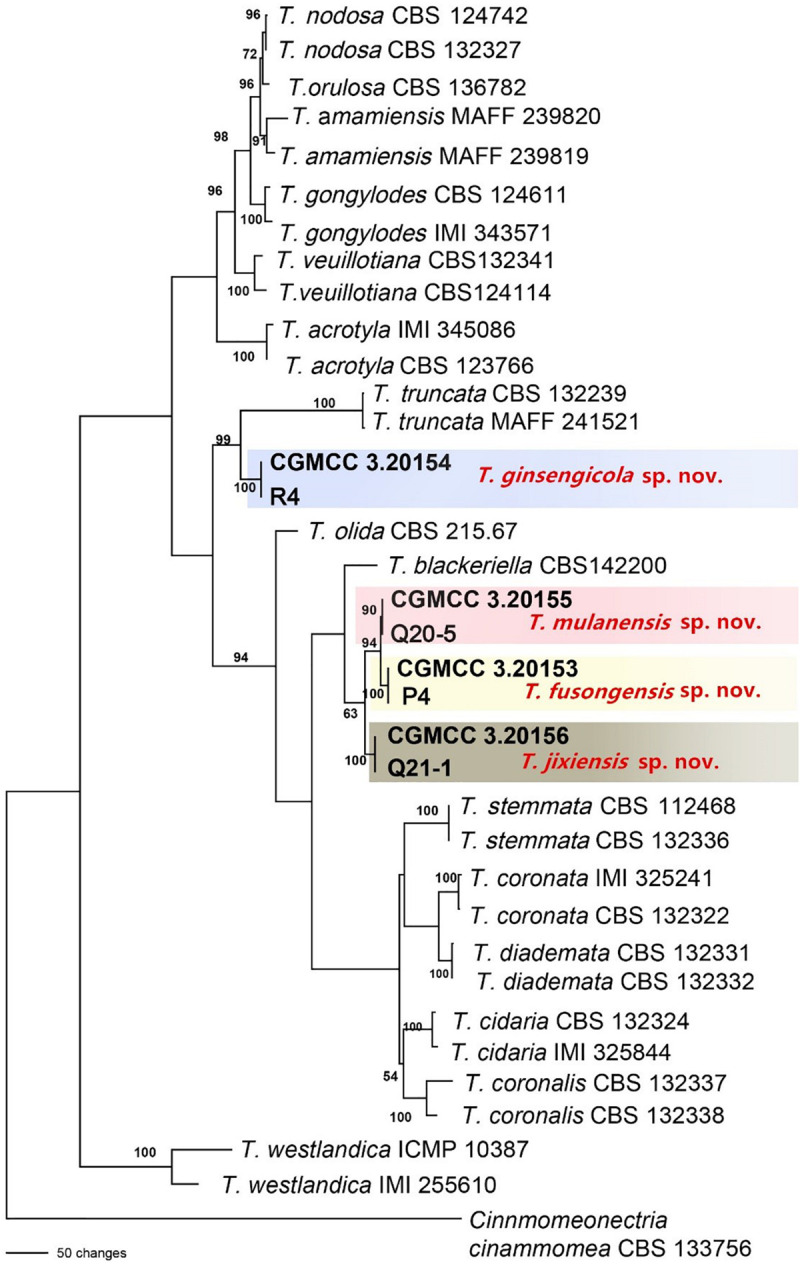
Phylogenetic tree based on the combined *ACT*, rDNA-ITS, LSU, *RPB1*, *RPB2*, SSU, *TEF* and *TUB* gene sequences constructed using the maximum parsimony method of the PAUP 4.0b program. *Cinnmomeonectria cinammomea* CBS 133756 was used as an outgroup. Bold font indicates the strains isolated in this study.

A total of 766 isolates were obtained from ginseng roots that showed typical signs of rusty root rot disease. All of these strains were determined by sequencing and morphological characteristics, and 26 strains from each genus were randomly selected for phylogenetic analysis. A total of 14 species (Figures 3, 4) were identified as *Ilyonectria*, *Ilyonectria*-like and *Rhexocercosporidium* species.

The *Ilyonectria* species complex included *I. robusta*, *I. communis*, *I. mors-panacis*, *I. pseudodestructans*, *I. changbaiensis* and *I. qitaiheensis*, and the isolate proportions were 55.0%, 21.7%, 10.9%, 2.0%, 1.3% and 1.3%, respectively. *Ilyonectria*-like contained three genera that were also members of the Nectriaceae family: *Dactylonectria*, *Neonectria* and *Thelonectria*. *N. obtusispora, D. torresensis* and *D.* sp. accounted for 2.0%, 0.5% and 0.5% of the population, respectively. Four novel species are named in this article: *T. ginsengicola* (1.0%), the type strain is R9; *T. jixiensis* (1.0%), the type strain is Q21-5; *T. mulanensis* (0.8%), the type strain is Q20-8 and *T. fusongensis* (0.5%), the type strain is R1-8. *R. panacis* was the only pathogen except *Ilyonectria*-like that was present at an abundance of 1.5%.*I. communis, I. pseudodestructans, I. changbaiensis, I. qitaiheensis, N. obtusispora, D. torresensis, T. jixiensis*, *T. mulanensis*, *T. fusongensis* and *T. ginsengicola* were first reported to cause ginseng rusty root rot. *Thelonectria* is also the first *Ilyonectria*-like genus reported to cause ginseng rusty root rot. *I. robusta* was the dominant pathogen and appeared in the largest proportion; this is the greatest difference between our results and previous conclusions. The number of fungal isolates recovered from each sampling location in China is shown in Table 4.

**TABLE 4:** Fungal isolates recovered from *Panax ginseng* with rusty root rot symptoms in China.

**Location**	**A**	**B**	**C**	**D**	**E**	**F**	**G**	**H**	**I**	**J**	**K**	**L**	**M**	**N**
Jindou Town, Tonghua City, Jilin Province, China	12	2								1				
Chaoyang Forestry Centre, Tonghua City, Jilin Province, China	7		2							2				
Sanyuanpu Town, Jian City, Jilin Province, China	9	6						1						
Duling Town, Tonghua City, Jilin Proinvce, China	5							2						
Fujiang Town, Tonghua City, Jilin Province, China	4	4								1				
Jiangdianzi Town, Tonghua City, Jilin Province, China	6		2											
Qinghe Town, Jian City, Jilin Province, China	10													
Yulin Town, Jian City, Jilin Province, China	7	3								2				
Shuangcha Town, Jian City, Jilin Province, China	8													
Maxian Town, Jian City, Jilin Province, China	6													
Linghou Town, Jian City, Jilin Province, China	7	5	1											
Taishang Town, Jian City, Jilin Province, China	11							1						
Sankeyushu Town, Jian City, Jilin Province, China	6													
Zuojia Town, Jili City, Jilin Province, China	3			6										
Longfeng Forestry Centre, Jiaohe City, Jilin Province, China	3													
Wulin Town, Jiaohe City, Jilin Province, China	4													
Qianjin Town, Jiaohe City, Jilin Province, China	6													
Dongbeicha Town, Baishan City, Jilin Province, China	17	12	3								3			
Longquan Town, Baishan City, Jilin Province, China	8	11	4		2	2	4			1				
Xingshen Town, Baishan City, Jilin Province, China	19	3	1											
Beigang Town, Baishan City, Jilin Province, China	11	9	4		1		1			1				
Quanyang Town, Baishan City, Jilin Province, China	10	6	5		1						1			
Erdaogang Town, Baishan City, Jilin Province, China	17	8	3			2								
Donggang Town, Baishan City, Jilin Province, China	13	5	5				2							
Xigang Town, Baishan City, Jilin Province, China	7	5	6		2									
Fusong County, Baishan City, Jilin Province, China	6	12				2					3			2
Wanliang Town, Fusong County, Baishan City, Jilin Province, China	2	6	4											2
Toudao Town, Helong City, Jilin Province, China	3	5												
Datougou Town, Wangqing City, Jilin Province, China	7	2		2	1									
Fuxing Town, Wangqing City, Jilin Province, China	8													
Luozigou Town, Wangqing City, Jilin Province, China	6													
Manzu Town, Hunchun City, Jilin Province, China	5	3	5	2										
Yangpao Town, Hunchun City, Jilin Province, China	4		3											
Yingan Town, Dunhau City, Jilin Province, China	8		6											
Qiuligou Town, Dunhau City, Jilin Province, China	4	5		3										
Dashitou Town, Dunhau City, Jilin Province, China	9													
Yongqing Town, Antu City, Jilin Province, China	11								2					
Yongqing Town, Antu City, Jilin Province, China	2	2		2										
Xinhe Town, Antu City, Jilin Province, China	9								2					
Fuxing Town, Fuxin City, Liaoning Province, China	10													
Huanren Town, Huanren City, Liaoning Province, China	3													
Xinbin Town, Xinbin City, Liaoning Province, China	2									1				
Hulin Town, Jixi City, Heilongjiang Province, China	5	5	3		1							3		
Hulin Town, Jixi City, Heilongjiang Province, China	5	2	6		2							5		
Hailin Town, Mudanjiang City, Heilongjiang Province, China	8	4					2							
Ningan Town, Mudanjiang City, Heilongjiang Province, China	6	2	1							1				
Dongjing Town, Mudanjiang City, Heilongjiang Province, China	3	1	1				3							
Yongshun Town, Qitaihe City, Heilongjiang Province, China	6	5	3							1				
Hailun Town, Hailun City, Heilongjiang Province, China	11	4	6											
Binzhou Town, Haerbin City, Heilongjiang Province, China	2	2	2			1								
Bayan Town, Mulan County, Heilongjiang, China	9	3	3				1						3	
Mulan Town, Mulan County, Heilongjiang, China	9	5	4										3	
Shangzhi Town, Shangzhi City, Heilongjiang, China	8	6				2								
Zhanhe Town, Heihe City, Heilongjiang, China	11	8				1	2							
Qingan Town, Suihua City, Heilongjiang, China	12													
Tieli Town, Yichun City, Heilongjiang, China	11	5	1											
Total	421	166	84	15	10	10	15	4	4	11	8	8	6	4

*A, *I. robusta*; B, *I. communis*; C, *I. mors-panacis*; D, *I. pseudodestructans*; E, *I. changbaiensis*; F, *I. qitaiheensis*; G, *N. obtusispora*; H, *D. torresensis;* I, *D. sp.*; J, *R. panacis*; K, *T. ginsengicola;* L, *T. jixiensis*; M, *T. mulanensis*; N, *T. fusongensis.**

### Molecular Phylogenetic Analysis

The sequences of rDNA-ITS, *TEF*, *TUB* and *HIS*3 were obtained using polymerase chain reaction amplicons and were analyzed in *Dactylonectria*, *Ilyonectria*, and *Neonectria*. Low-quality sequences were removed after base alignment to obtain the sequences of rDNA-ITS (491 bp), *HIS3* (472 bp), *TEF* (507 bp), and *TUB* (467 bp). The sequences were combined, and a sequence with a total length of 2231 bp, including alignment gaps, was used to build a phylogenetic tree (Figure 3). Similarly, *ACT* (575 bp), rDNA-ITS (533 bp), LSU (700 bp), *RPB1* (630 bp), *RPB2* (856 bp), SSU (521 bp), *TEF* (858 bp), and *TUB* (479 bp) were combined, resulting in a total length of 4422 bp including alignment gaps, and analyzed for *Thelonectria* (Figure 4). All of the sequences were topologically congruent, and the results indicated that *Dactylonectria*, *Ilyonectria*, *Neonectria and Thelonectria* formed a single clade. The isolated strains were divided into 14 highly supported clades; the branches containing *I. rubosta, I. mors-panacis* and other known species were highly consistent with species type or verified species.

Four novel species were divided into independent clades, and they were supported with 90-100% bootstrap support for *T. mulanensis*, *T. jixiensis*, *T. fusongensis* and *T. ginsengicola* (Figure 4). For *D.* sp., we observed only a few macroconidia, and the specific sporulation structure and other morphological characteristics were not clear. Therefore, the species is not named here. Similarly, a separate gene phylogenetic tree was constructed for *Thelonectria*, with the least information obtained from the SSU and *TUB* genes, and the lowest contribution. *ACT* and *RPB2* were the most informative and were key to distinguishing the phylogenetic tree of all of the known candidate strains and four novel species. The combined use of rDNA-ITS, *TUB*, *TEF*, and LSU genes will aid in the classification and analysis of all candidate species. The four novel species of *Thelonectria* belong to the *T. coronata* complex classification reported in 2016 (Salgado-Salazar et al., 2016). The three closely related species *T. mulanensis, T. fusongensis*, and *T. jixiensis* have nucleotide sequence differences in their *ACT*, rDNA-ITS, LSU, *RPB1*, *RPB2*, SSU, *TEF* and *TUB* genes (Table 5). There is no nucleotide difference in the SSU gene, 1 bp difference in the rDNA-ITS and LSU genes, and 2 bp differences in the *RPB1* gene; thus, these three genes are ineffective for distinguishing the three species. These three species have 5 bp differences in *ACT*, 14 bp differences in *RPB2*, 19 bp differences in *TEF* and 4 bp differences in *TUB*. *T. mulanensis* and *T. fusongensis* have 3 bp differences in *RPB2*, 5 bp differences in *TEF* and 4 bp differences in *TUB*. Phylogenetic analysis combining the *RPB2*, *TEF* and *TUB* genes is most effective at distinguishing *T. mulanensis* and *T. fusongensis*. *T. mulanensis* and *T. fusongensis* were clustered on a branch with a close genetic relationship and may form a complex group. However, due to their distinct morphological characteristics of conidia size and number of septa, they were considered two novel species.

**TABLE 5:** Nucleotide differences in the partial gene sequences of rDNA-ITS, *ACT*, LSU, *RPB1*, *RPB2*, *TEF*, and *TUB* for *T. mulanensis*, *T. fusongensis* and *T. jixiensis.*

**Species**	**Position (bp)**																																			
	**rDNA-ITS**			***ACT***						**LSU**	***RPB1***		***RPB2***																							
	141			1	223	226		334	343	36	370	400	9	12		13		20		21	26		27		28		29	30		31		57		687		699
***T. mulanensis***	C			T	T	C		C	C	–	G	G	A	C		A		G		C	C		–		C		A	T		C		G		T		G
***T. fusongensis***	C			T	T	C		T	T	–	G	G	G	C		A		G		C	C		–		C		A	T		A		G		C		G
***T. jixiensis***	T			C	C	T		C	C	A	A	C	A	T		C		C		A	G		A		T		C	C		A		T		C		T

**Species**	**Position (bp)**																																			
	***TEF***																													***TUB***						
	23	52	72	134	164	167	175	195	372	403	411	416	419		423		424		448			558		788		833			59		60		300		318	

***T. mulanensis***	C	A	C	–	G	–	G	–	C	A	C	T	A		C		A		T			C		T		C			G		T		G		T	
***T. fusongensis***	T	A	G	A	G	–	G	–	C	A	T	C	A		C		A		T			C		C		C			A		C		C		C	
***T. jixiensis***	T	T	C	–	A	A	A	T	T	G	T	C	C		–		–		C			T		T		T			G		T		G		T	

*Position refers to the nucleotide position in each sequence of rDNA-ITS, *ACT*, LSU, *RPB1*, *RPB2*, *TEF* and *TUB* of type isolates.*

### Brief Introduction to the Morphological Characteristics of *Ilyonectria* Species

Morphological characteristics also support the classification of phylogenetic trees. The morphological characteristics of *I. robusta, I. communis, I. mors-panacis, I. pseudodestructans, I. changbaiensis, I. qitaiheensis, N. obtusispora*, *D. torresensis* and *R. panacis* were consistent with previously reported strains. Four novel species were confirmed by phylogeny and are described as follows.

***Thelonectria ginsengicola*** Y. M. Guan & Y. Li, sp. nov.

**FIGURE 5:**
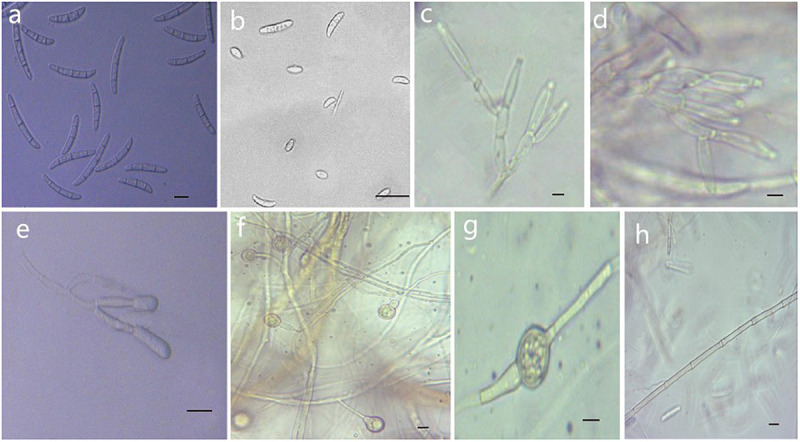
Morphological characteristics of *Thelonectria ginsengicola* (CGMCC3.20154). **(a)** Macroconidia. **(b)** Microconidia. **(c-e)** Conidiophores. **(f,g)** Chlamydospores. **(h)** Hypha. Bar = 10 μm.

MycoBank MB836525 (Figure 5).

**Etymology:** Named after the host *Panax ginseng*. “*Ginsengicola*” means “born on ginseng.”

**Type:** China, Jilin Province, Changbai City, county of Fusong, on roots of *Panax ginseng*, Oct 2018, Y. Guan. (CGMCC3.20154 = R9 - holotype).

**Description:** *Sexual morph*: Undetermined. *Asexual morph*: Conidiophores were simple or complex with 2-3 branches, monophialides. Mycelial cells were thick-walled and septate. Hyphae hyaline to yellow. Chlamydospores were borne apically or intermittently in hyphae and were more commonly single or in pairs. Macroconidia were the majority, with 1-5 septa; most had 3-4 septa. The shape was a sickle-shaped curve; the base foot was blunt and became gradually thicker toward the top, and the top was also blunt. The size was (38.4) 44.3 (57.7) μm × (4.8) 5.7 (6.9) μm, and the length/width ratio was (5.9) 8.1 (10.3). Microconidia were elliptical, stick or sickle-shaped curves with an irregular shape and 0-1 septa. The size of microconidia was (12.5) 9.69 (12.5) μm × (4.8) 4.1 (6.9) μm, and the length/width ratio was (1.4) 2.7 (3.7). The size of one septate microconidium was (19.5) 24.3 (29.3) μm × (4.1) 4.8 (6.5) μm, and its length/width ratio was (3.9) 5.5 (6.4).

**Culture characteristics:** On PDA, the color was initially white, later becoming dark yellow with a wavy margin; the reverse side was reddish brown. The colony grows outward in a wave shape. The mycelium grew vigorously, and it was difficult to produce conidia on PDA and SNA. After the addition of ginseng roots and continuous n-UV irradiation, pionnotes were produced on the mycelium or on the vertical wall of the petri dish. The growth of the colony formed concentric rings, and the growth was very slow. The size of the colony reached 22 mm in 10 days. On SNA, the colonies were sparse and white to transparent with regular margins and chlamydospores; macroconidia and microconidia were not observed.

**Note:** *T. ginsengicola* and *T. truncata* were grouped together on a branch. The greatest difference was the presence of microconidia and chlamydospores. Macroconidia were smaller than those of *T. truncate* (Salgado-Salazar et al., 2012).

***Thelonectria jixiensis*** Y. M. Guan & Y. Li, sp. nov.

**FIGURE 6:**
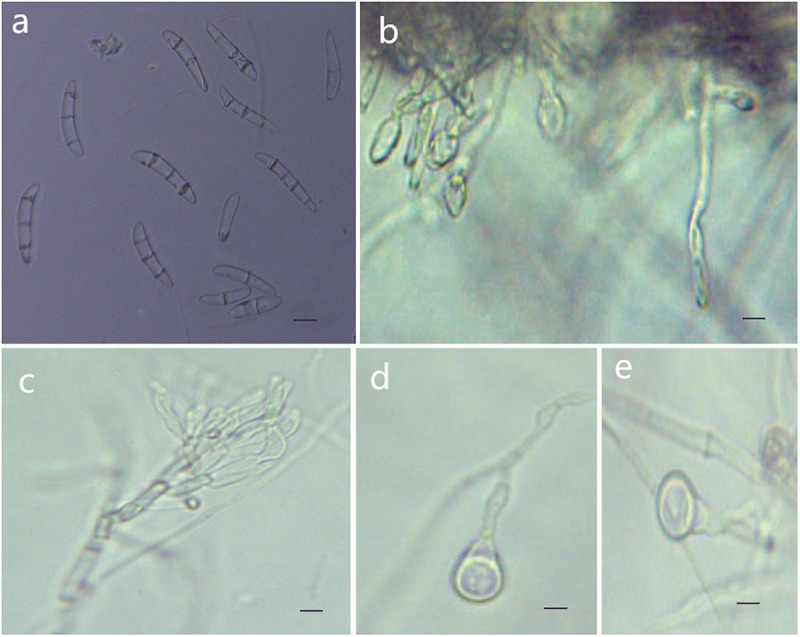
Morphological characteristics of *Thelonectria jixiensis* (CGMCC3.20156). **(a)** Macroconidia. **(b)** and **(c)** Conidiophores. **(d)** and **(e)** Chlamydospores. Bar = 10 μm.

MycoBank MB 836520 (Figure 6).

**Etymology:** Named after the city of Jixi, Heilongjiang Province, China, where the strain type was collected.

**Type:** China, Heilongjiang Province, Jixi City, on roots of *Panax ginseng*, Oct 2018, Y. Guan. (CGMCC3.20156 = Q21-5 - holotype).

**Description:** *Sexual morph*: Undetermined. *Asexual morph*: Conidiophores simple or complex, spiral branches, monophialides and straight macroconidia or slightly curved head. The body is widest at one-quarter of the head; the basal cells with tips are thinner and narrower, with 0-4 septa. Most had 2-3 septa, and very few had 0 or 4 septa; sizes were in the range of (27.3) 35.8 (41.7) μm × (5.5) 6.9 (8.9) μm, with a length/width ratio of (4.3) 5.1 (7.9). Microconidia were not observed. Chlamydospores were mostly borne apically on the mycelium, and solitary was more common.

**Culture characteristics:** On PDA, the colonies produced were white at the beginning with a sparsely floccose to fluffy aerial mycelium and vigorous growth. The hyphae at the margins of the colony were thin, and the center hyphae were denser. Light yellow color appeared occasionally on the reverse side over time. The colony grew slowly to a size of 38 mm in 10 days. On SNA, the colonies were sparse, with regular margins, and the mycelium obviously degenerated and gradually disappeared as the number of breeding generations increased. Chlamydospores and macroconidia were not observed on SNA.

**Note:** The phylogenetic tree shows that *T. jixiensis* is closely related to *T. blackeriella*. *T. jixiensis* produced simple or complex conidiophores with spiral branches, and its macroconidia were slightly larger. The simple conidiophores of *T. blackeriella* and the macroconidia were relatively small. The shapes of the macroconidia of the two are very easily distinguished, and neither species produces microconidia (Carlucci et al., 2017).

***Thelonectria mulanensis*** Y. M. Guan & Y. Li, sp. nov.

**FIGURE 7:**
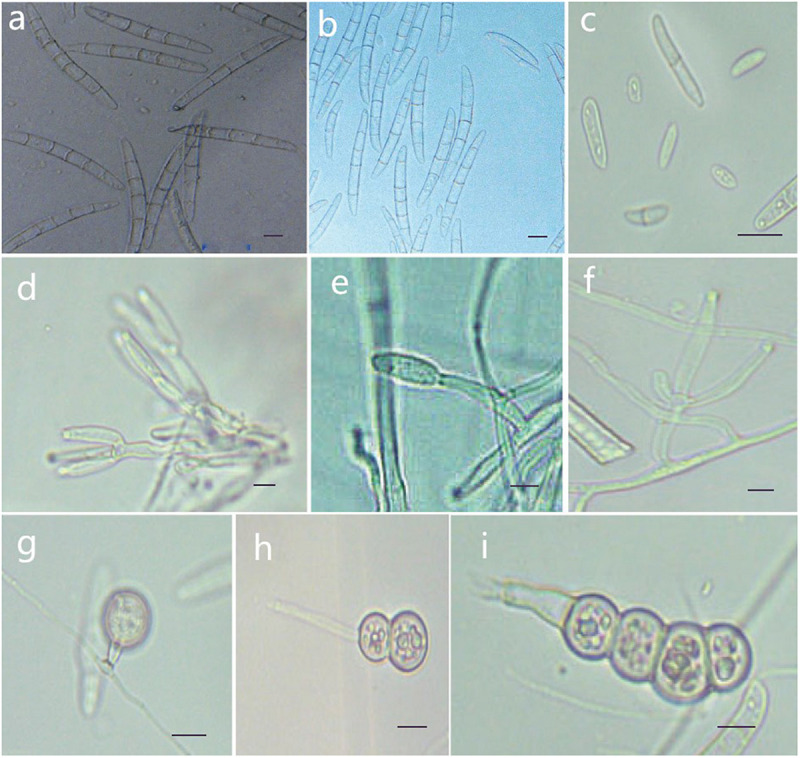
Morphological characteristics of *Thelonectria jixiensis* (CGMCC3.20155). **(a,b)** Macroconidia. **(c)** Microconidia. **(d–f)** Conidiophores. **(g–i)** Chlamydospores. Bar = 10 μm.

MycoBank MB 836521 (Figure 7).

**Etymology:** Named after the County of Mulan, Heilongjiang Province, China, where the strain type was collected.

**Type:** China, Heilongjiang Province, Mulan County, on roots of *Panax ginseng*, Oct 2018, Y. Guan. (CGMCC3.20155 = Q20-8 - holotype).

**Description:** *Sexual morph*: Undetermined. *Asexual morph*: Conidiophores simple or complex, with 2-3 branches, monophialides. The mycelial cells were thick-walled, transparent and septate. The chlamydospores were produced from the mycelium, and spores were intercalary or terminal and single, in pairs or multiple bunches. Macroconidia were the majority, with 3-7 septa, but most were 5 septa, and lengths ranged from (59.1) 76.5 (96.6) μm × (2.7) 8.3 (5.6) μm. Fungi were a sickle-shaped curve. The base foot was blunt and became gradually thicker toward the top, and the top was also blunt. The microconidia were elliptical, stick or sickle-shaped curved of an irregular shape with 0-1 septum, and lengths ranged from (4.6) 9.5 (18.3) μm × (2.7) 3.9 (5.6) μm. Microsclerotia were produced after more than 8 weeks.

**Culture characteristics:** On PDA, the colonies produced a white, cottony dense floccose to fluffy aerial mycelium. The margins of the colony were irregular, and the hyphae were sparse and radial. The reverse color was white to yellow. In PDA culture, colonies grew slowly and reached 25 mm in 10 days. On SNA, the colonies were sparse, white to transparent, with regular margins and chlamydospores; macroconidia and microconidia were not observed.

**Note:** This species is closely related to *T. jixiensis*, but it produces macroconidia and microconidia, and *T. jixiensis* has no microconidia. The macroconidia of *T. mulanensis* were twice the size of those of *T. jixiensis*, had more septa and were thinner. The conidiophores and chlamydospores of the two species are similar.

***Thelonectria fusongensis*** Y. M. Guan & Y. Li, sp. nov.

**FIGURE 8:**
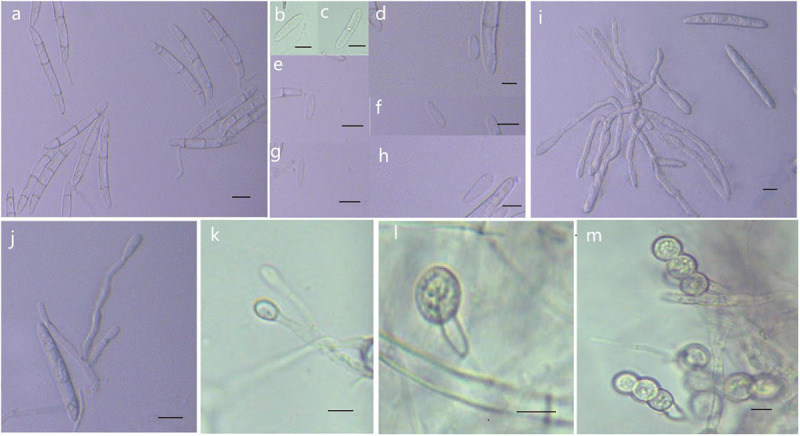
Morphological characteristics of *Thelonectria jixiensis* (CGMCC3.20154). **(a)** Macroconidia. **(b–h)** Microconidia. **(i–k)** Conidiophores. **(l,m)** Chlamydospores. Bar = 10 μm.

MycoBank MB 836524 (Figure 8).

**Etymology:** Named after the county of Fusong, Changbai City, Jilin Province, China, where the strain type was collected.

**Type:** China, Jilin Province, Changbai City, county of Fusong, on roots of *Panax ginseng*, Oct 2018, Y. Guan. (CGMCC3.20153 = R1-8 - holotype).

**Description:** *Sexual morph*: Undetermined. *Asexual morph*: Conidiophores were simple or complex, with 2-3 branches, monophialides. The mycelial cells were thick-walled, transparent, and septate. Chlamydospores were borne apically or intercalary in hyphae and were solitary or in pairs. Macroconidia were in the majority, with 1-5 septa, but most had 2-3 septa. The body was a sickle-shaped curve, and the base was blunt and thickened gradually toward the top. The top was also blunt, not sharp, with sizes in the range of (40.2) 52.3 (62.2) μm × (4.2) 5.9 (7.9) μm. Microconidia were in the minority and exhibited irregular elliptical, stick or curved sickle shapes and 0-1 septa, with sizes in the range of (7.4) 13.5 (23.3) μm × (2.9) 4.1 (6.1) μm.

**Culture characteristics:** On PDA, the colonies grew in a concentric wavy pattern and presented a velvety surface; they were initially white, then brownish yellow, and brownish red on the reverse side. The mycelium became increasingly sparse from the center to the edge. It was difficult to produce conidia on PDA and SNA. The addition of ginseng roots and continuous n-UV light cultivation caused the surface of the mycelium to produce yellow pionnotes at more than 8 weeks. The colonies reached 30 mm in diameter in 10 days. On SNA, the colonies were sparse, white to transparent, with regular margins and chlamydospores; macroconidia and microconidia were not observed.

**Note:** Phylogenetic inference revealed that *T. fusongensis* is closely related to *T. mulanensis*, but the microconidia of the former were larger than those of the latter, there were fewer septa, the microconidia were straighter, and the bending arc was smaller.

### Pathogenicity Tests

**FIGURE 9:**
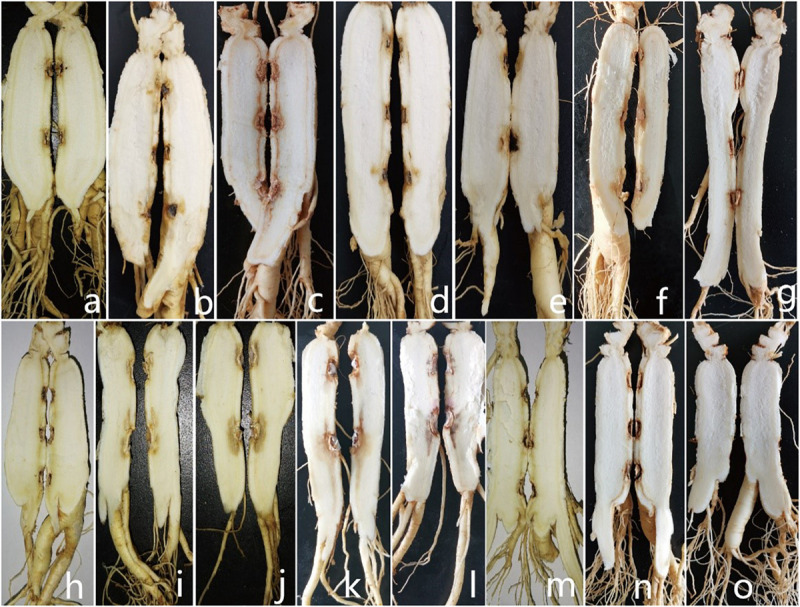
Symptoms displayed by 3-year-old ginseng roots inoculated with pathogens (longitudinal sections taken at the inoculation centerline). **(a-n)** *I. robusta* (H8-5), *I. communis* (CB4-2), *I. mors-panacis* (H6-1), *I. pseudodestructans* (ZP2), *I. changbaiensis* (CB4-7), *I. qitaiheensi* (R3-2), *N. obtusispora* (H7-6), *D. torresensis* (DT2), *D.* sp. (Q18-22), *T. ginsengicola* (CGMCC 3.20154), *T. jixiensis* (CGMCC 3.20156), *T. mulanensis* (CGMCC 3.20155), *T. fusongensis* (CGMCC 3.20153), and *R. panacis* (RP17). **(o)** control.

For *in vitro* inoculation using spore suspension, evaluation after 7 days showed that all of the strains infected ginseng roots. The mycelium grew on the ginseng root, and the longitudinal portion of the ginseng root also showed infection. Reddish-brown areas formed on the shallow ginseng roots near the inoculation point. The control showed no symptoms (Figure 9).

**FIGURE 10:**
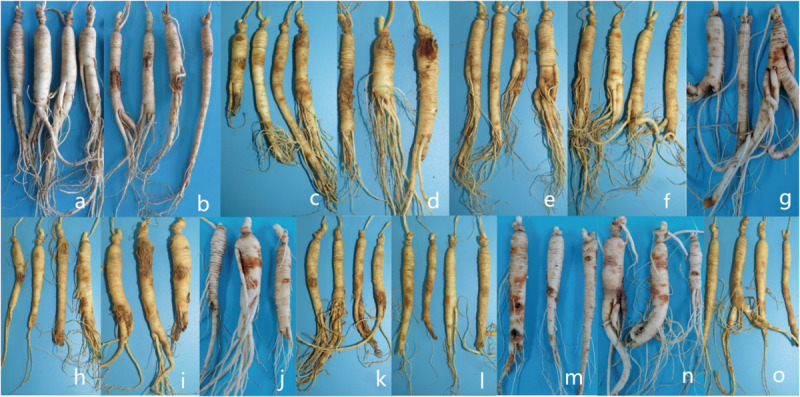
Symptoms displayed by 3-year-old ginseng roots inoculated with pathogens under greenhouse conditions. **(a)** control. **(b-o)** *I. robusta* (H8-5), *I. communis* (CB4-2), *I. mors-panacis* (H6-1), *I. pseudodestructans* (ZP2), *I. changbaiensis* (CB4-7), *I. qitaiheensi* (R3-2), *N. obtusispora* (H7-6), *D. torresensis* (DT2), *D.* sp. (Q18-22), *T. ginsengicola* (CGMCC 3.20154), *T. jixiensis* (CGMCC 3.20156), *T. mulanensis* (CGMCC 3.20155), *T. fusongensis* (CGMCC 3.20153), and *R. panacis* (RP17).

For whole plant inoculation in the greenhouse, all of the strains used in the test caused rusty root rot. 2 months after inoculation with the spore suspension, the inoculated 3-year-old ginseng roots showed symptoms similar to rusty root rot and to natural disease in the field. Some ginseng roots were rust-colored, but no gully like symptoms formed (Figure 10). Ginseng roots inoculated with sterile water were asymptomatic. Recovery of the diseased samples and sequencing of all of the genes described above yielded results that were the same as those obtained for the isolates used in the inoculations, satisfying Koch’s postulates, and the identity of the species was determined. Ginseng roots inoculated with *R. panacis* showed a darker color with grayish-black symptoms.

## Discussion

Ginseng rusty root rot is the root disease with the highest incidence in China’s main ginseng cultivation areas. The identities of the varieties of diseased ginseng samples collected in the experiments were not clear. Most of the currently grown Chinese cultivars are domesticated varieties, and Damaya ginseng is commercially cultivated. Therefore, the correlation between the presence of fungi on ginseng roots and ginseng variety was not analyzed. The identification and pathogenicity analysis of 766 isolates revealed that ginseng rusty root rot was caused by a complex of *Ilyonectria*, *Ilyonectria*-like, and *Rhexocercosporidium* fungi. The *Ilyonectria*-like fungi included three genera of *Ilyonectria*, *Dactylonectria* and *Thelonectria*, all of which belonged to Nectriaceae, except *Rhexocercosporidium*. *I. robusta* and *I. communis* were the dominant pathogens with the highest proportion of ginseng rusty root rot pathogens that were recovered from ginseng rusty rot roots.

Ginseng rusty root rot has been attributed to the *Ilyonectria* fungus in China and South Korea (Wang, 2001; Farh et al., 2018). The 766 isolates we isolated were divided into 14 species, of which *R. panacis* was the only pathogen other than *Ilyonectria* and *Ilyonectria*-like species previously reported to cause rusty root rot symptoms. *I. robusta* was the most widely isolated dominant pathogen; this is inconsistent with a previous report that 90% of Chinese ginseng rusty root rot is caused by *I. radicicola*/*C. destructans* (Wang, 2001). *I. radicicola*/*C. destructans* may be a huge complex group in terms of morphological similarity (Cabral et al., 2012a). The species found later in various plants were not closely related to the original strain type CBS 264.5 (*I. destructans*), and *I.*/*C. destructans* has generally not been found to be as common as when it was first proposed and discovered.

Most of the *Ilyonectria* strains infecting ginseng were obtained from *P. quinquefolius*, and *P. ginseng* and *P. quinquefolius* differ significantly with respect to the proportions of pathogens present. *I. crassa* and *I. panacis* were isolated from *P. quinquefolius* in Canada (Cabral et al., 2012a). The sampling collections of the present study covered the main cultivated areas of Chinese ginseng, and *I. panacis*, *I. rufa* and *I. crassa* strains were not isolated. In contrast, the dominant pathogen *I. robusta* was once considered to exhibit low pathogenicity. The pathogenicity was not directly proportional to the separation ratio. *I*. *robusta* isolated from *P. quinquefolium* was first described by Hildebrand as *Ramularia robusta* (Hildebrand, 1935). *I. robusta* was first reported in 2014 to cause ginseng root disease in China (Lu et al., 2014). Sixty-eight strains were derived from many hosts. Twenty-one of the isolates obtained from ginseng plants were segregated phylogenetically into four different groups, each of which was considered a different species of *Ilyonectria*: *I. mors-panacis*, *I. robusta*, *I. panacis*, and *I. crassa* (Seifert et al., 2003; Cabral et al., 2012a). These strains were grouped according to the strength of their pathogenicity; the weakly aggressive strains also originated from other hosts, but the highly aggressive strains were derived from a specific host ginseng (Cabral et al., 2012a). *I. mors-panacis* is not limited to infecting ginseng (Mi et al., 2017). *I. robusta* is considered a weakly aggressive strain based on the phylogenetic analysis and on *I. robusta’*s and *I. crassa*’s host and other factors. It is speculated that American and Korean ginseng are not susceptible to *I. robusta* or *I. crassa* (Cabral et al., 2012a), and the present article confirmed that *I. robusta* was the dominant pathogen with the highest isolation ratio. The prevention and control of rusty root rot in cultivation should be dominated by attention to *I. rubosta*.

Ginseng red-skin root is a non-infectious disease that differs from ginseng rusty root rot in China. Lu et al. reported that ginseng red-skin root is caused by a complex group of *Fusarium*, *Ilyonectria* and *Dactylonectria* and that *Ilyonectria* fungi were the dominant pathogens. *I. communis*, *I. changbaiensis* and *I. qitaiheensis* were newly reported species, but the same species were obtained in the present study. The types of pathogenic populations obtained in the present research are similar to the previously identified pathogens. Red-skin root and rusty root rot may not be separated. These conditions are primarily caused by *Ilyonectria* and *Ilyonectria*-like fungi, and they may represent different infection stages or may be caused by different species of *Ilyonectria* and *Ilyonectria*-like fungi (Lu et al., 2020). However, this hypothesis must be clarified. The uniform name “rusty root rot” is more acceptable because most of the infected ginseng roots have dry rot that is similar to the symptoms of decayed wood, not merely red skin; it is possible that the pathogens that cause rusty root rot also cause red-skin root and that red-skin roots are only in the early stages of the disease. *I. communis* and *I. robusta* were also the two pathogens present in the highest proportions in red-skin root isolates. Differences in pathogenicity may be the most widely accepted cause of the different symptoms, but if the disease is inferred based only on the apparent form of the agents infecting the ginseng root, the diagnosis will not be acceptable. The lifestyle of each microorganism is different, and different effects may not result from differences in pathogenicity. *Fusarium* was not reported as a pathogen that causes dry rot symptoms alone. *Fusarium* may infect the roots of ginseng via multi-infection in tissues infected by primary intruders that are not the direct cause of rusty root rot or red-skin root (Guan et al., 2014; Gao et al., 2014; Wang et al., 2016).

*I. mors-panacis* was discovered on *P. quinquefolium* by Hildebrand (1935), who described it as “*R. mors-panacis*” (Hypocreales, 1935). This species was also found in Japan on *P. ginseng*, and it was collected as “*C. destructans*” f. sp. *Panacis* (ex-type CBS 124662) is treated as a synonym. This species is considered a strong, aggressive species in *Ilyonectria*, but the isolation ratio was only 10.9%. Sufficient time to complete the infection is necessary for the chronic pathogenic *Ilyonectria* population to cause damage to the roots of perennials (Guan et al., 2020). Of course, the relationship between the degree of epidemic disease and pathogenicity was not studied in depth, and this relationship is determined by many factors. Although *I. communis* is a new species that was only recently reported (Lu et al., 2020), it is one of the dominant pathogens that cause ginseng rusty root rot. *I. robusta* and *I. communis* should be the main targets for controlling ginseng rusty root rot in cultivation.

The four novel species of *Thelonectria* were identified based on the conclusion that closely related species were being compared. Multi-locus studies are feasible and effective for the identification of novel species. None of the individual genes studied completely matched the same gene in another species, and the combined phylogenetic analysis of multiple gene sequences may result in a suitable classification. Among the studied genes, SSU contributed very little to the strains listed in the present article, but its role cannot be ignored in *Thelonectria* (Salgado-Salazar et al., 2016). *ACT* and *RPB2* provided the most information, and analysis of these genes could fully resolve all of the strains described in this article. Comparison of the *RPB2*, *TEF* and *TUB* genes is the most effective way to distinguish *T. mulanensis* from *T. fusongensis*. The use of all of the gene sequences studied in this work makes the phylogenetic tree more credible. For *Ilyonectria*, rDNA-ITS was not a good marker for distinguishing strains such as *I. rufa* and *I. robusta*, and the *HIS3* gene may be used alone to initially distinguish species of *Ilyonectria* (Cabral et al., 2012b).

Many novel *Ilyonectria* and *Ilyonectria*-like species have been discovered recently (Carlucci et al., 2017; Lawrence et al., 2019). Due to the slow growth of some fungi, it is not easy to obtain species using separation techniques. Novel species of *Ilyonectria* and *Ilyonectria*-like fungi that infect the roots of ginseng will continue to be discovered in soil. *Thelonectria* fungi have saprophytic properties or weak pathogenicity, and their host specificity has not been determined. Evaluation after inoculation also showed that *Thelonectria* fungi had weak pathogenicity, and these fungi may also be opportunistically pathogenic, similar to *Ilyonectria*-like fungi.

The present research revealed pathogens that cause ginseng rusty root rot but have remained unknown for many years. These results, which were obtained using a multi-locus combined approach, update previous conclusions and identify the species that should be targeted for the prevention and control of rusty root rot in ginseng cultivation. However, due to the continuous cropping properties of ginseng and continuous land use changes, *Ilyonectria* and *Ilyonectria*-like pathogens are soil-borne diseases, and the population structure may also change.

## Data Availability Statement

The datasets presented in this study can be found in online repositories. The names of the repository/repositories and accession number(s) can be found below: https://www.ncbi.nlm.nih.gov/genbank/, MT678559 etc.

## Author Contributions

YG conceived and designed the study and wrote the manuscript. YG, YM, and QJ performed the experiments. QW, NL, and YF analyzed the sequences. YZ and YL reviewed and edited the manuscript. All authors read and approved the manuscript.

## Conflict of Interest

The authors declare that the research was conducted in the absence of any commercial or financial relationships that could be construed as a potential conflict of interest.
